# The Student’s Medical Dictionary

**Published:** 1896-12

**Authors:** 


THE STUDENT'S MEDICAL DICTIONARY. Including all the words and phrases generally used in medicine. with their proper pronunciation and definitions. Based on recent medical literature. By GEORGE M. GOULD, A. M., M. D., author of An illustrated dictionary of medicine, biology and allied sciences, etc., etc. With elaborate tables of the bacilli, micrococci, leucomains, ptomains, etc. ; of the arteries, ganglia, muscles and nerves ; of weights and measures, etc., etc. Tenth edition, rewritten and enlarged. Pages 701. Philadelphia : P. Blakiston, Son & Co., 1012 Walnut street. 1896.

In the preface to the tenth edition the author states that the volume at hand is especially adapted to the wants of medical students and should be regarded rather as an introduction than a substitute for the larger illustrated work. This volume nicely fills the gap, and to become acquainted with this dictionary is to prize it very highly. It is perhaps just as well to omit illustrations in a dictionary, as they must, of necessity, be small and hence incomplete. A really good dictionary is one which in a small space gives you dictionary information regarding the word you are seeking, its pronunciation and spelling. When a dictionary does more than this it becomes an encyclopedia and should be labeled such.

Dr. Gould has made an excellent dictionary out of this volume, and to those who possess an encyclopedia of some kind it serves as a most instructive introduction. The tables, especially of the eponymic diseases, the lines or lineæ, the ganglia, the monstrosities, ptomains, poisons, reflexes, eponymic signs and symptoms of disease, are most valuable and save the busy practitioner much time and annoyance.

Dr. Gould has certainly found his forte in dictionary-making and is in a favorable position for carrying out much-needed reform in the spelling and pronunciation of many medical words.

